# Microbial Growth and Quorum Sensing Antagonist Activities of Herbal Plants Extracts

**DOI:** 10.3390/molecules14093425

**Published:** 2009-09-03

**Authors:** Reema Al-Hussaini, Adel M. Mahasneh

**Affiliations:** Department of Biological Sciences, Faculty of Sciences, University of Jordan, Amman 11942, Jordan; E-mail: reema_alhussaini@yahoo.com (R.A-H.)

**Keywords:** antimicrobial agents, antiquorum sensing, herbal extracts

## Abstract

Antimicrobial and antiquorum sensing (AQS) activities of fourteen ethanolic extracts of different parts of eight plants were screened against four Gram-positive, five Gram-negative bacteria and four fungi. Depending on the plant part extract used and the test microorganism, variable activities were recorded at 3 mg per disc. Among the Gram-positive bacteria tested, for example, activities of *Laurus nobilis* bark extract ranged between a 9.5 mm inhibition zone against *Bacillus subtilis* up to a 25 mm one against methicillin resistant *Staphylococcus aureus*. *Staphylococcus aureus* and *Aspergillus fumigatus* were the most susceptible among bacteria and fungi tested towards other plant parts. Of interest is the tangible antifungal activity of a *Tecoma capensis* flower extract, which is reported for the first time. However, minimum inhibitory concentrations (MIC's) for both bacteria and fungi were relatively high (0.5-3.0 mg). As for antiquorum sensing activity against *Chromobacterium violaceum*, superior activity (>17 mm QS inhibition) was associated with *Sonchus oleraceus* and *Laurus nobilis* extracts and weak to good activity (8-17 mm) was recorded for other plants. In conclusion, results indicate the potential of these plant extracts in treating microbial infections through cell growth inhibition or quorum sensing antagonism, which is reported for the first time, thus validating their medicinal use.

## Introduction

Control of microbial infections by inhibition of microbial growth or quorum sensing has been the base of antimicrobial chemotherapy [1]. However, an emerging problem associated with misuse of antibiotic therapy is the worldwide emergence of higher level tolerance of target organisms against available broad spectrum antibiotics [2]. As a result, and in the light of the rapid spread of multidrug resistance, the development of new antimicrobial or antipathogenic agents that act upon new microbial targets has become a very pressing priority [3]. In view of the fact that quorum sensing is involved in microbial pathogenesis, research efforts have focused recently upon developing antipathogenic agents to control bacterial diseases by inhibiting quorum sensing [4,5]. Antiqourum sensing agents would offer a way of controlling microbial infections with the advantage of reducing risks of resistance development [6]. The continuing search for new and novel antimicrobials and antipathogenic agents has focused on exploiting the fact that plants surviving in an environment with high bacterial density have been seen to possess protective means against infections [7]. Using this argument, researchers are increasingly looking at herbal products in the quest for new therapeutic and antipathogenic agents which might be nontoxic inhibitors of quorum sensing, thus controlling infections without encouraging the appearance of resistant bacterial strains [8]. Current literature estimates that 10% of all terrestrial flowering plants on earth have been used by different communities in treating diseases, however, only around 1% have gained recognition and validation [9].

Controlled studies indicate the great potential of phytochemicals to be the richest reservoir of new and novel therapeutics [10]. Although the antimicrobial activities of plant extracts are beyond doubt, in many instances their exact mechanism of antimicrobial functionality is not well understood [11,12,13].

Searching the literature, it is surprising to find very few works discussing plant extracts and their antiquorum sensing activities. It is believed that plant extracts with well documented antimicrobial activities could possess antipathogenic as well as antivirulent activities, which may not be linked to the growth and inhibition of the microorganism [1,14]. The antiquorum sensing activity of herbal plants is very poorly investigated and it is very likely that it will be found that the antimicrobial efficacy is mediated by quorum sensing inhibition. Due to the wide use of popular remedies originating from plants, the primary objective of this investigation was to determine the antiquorum, antibacterial and antifungal activities of the ethanolic extracts of different parts of eight different plants, in an effort to validate their use in folk medicine. These plants included *Tecoma capensis*, *Populus nigra*, *Populus alba*, *Sonchus oleraceus*, *Lavandula angustifolia*, *Rosmarinus officinalis*, *Laurus nobilis* and *Jasminum sambac*.

## Results and Discussion

Medicinal use of extracts obtained from plants in general have recently gained popularity, inducing scientific interest exemplified in screening programs for novel and new components and uses pertaining to microbial growth or bacterial quorum sensing inhibition [1,15].

To validate some aspects of the traditional uses of the tested plants (Table 1) as antibacterial and antifungal agents, extracts were tested against an array of Gram-positive, Gram-negative bacteria, filamentous fungi and yeasts. In addition, their antipathogenic potential was checked by examining the antiquorum sensing activity of such extracts using *Chromobacterium violaceum* assays.

**Table 1:** Ethnobotanical data about the studied plants.

Plant Scientific name,family, voucher numbers	Local name	Parts used	Ethnobotanical information: traditional and/or medicinal use	Preparation and administration
*Laurus nobilis* L. Lauraceae, MAHAS 1	El- ghar	Leaves	Condiment, flavoring Carminative, digestive problems Cold, bronchitis	Spiced meats Infusion/oral Vapor bath, decoction
		Fruits	Menstruation, earache, furunculosis Diuretic, anti- rheumatic	Raw berries, essential oils/ ointment Infusion/liniment
		Flowers	Food flavoring	As in leaves
		Bark	Food flavoring	As in leaves
*Populus alba* L. Salicaceae, MAHAS 2	Al-Hoor al-abyad	Leaves	Depurative, tooth decay	Decoction, Infusion/oral
*Populus nigra* L. Salicaceae, MAHAS 3	Al-Hoor al-aswad	Leaves	Tonic, antiseptic	Decoction/ external use
*Lavandula angustifolia* Mill. Lamiaceae, MAHAS 4	khuzama, lavender	Flowers	Bronchitis, cough Antiseptic	Infusion/oral Essential oils/ liniment
*Rosmarinus officinalis* L. Lamiaceae, MAHAS 5	Hasa-alban	Leaves Flowers	Antiseptic, antispasmodic, and food flavoring. Tonic, stimulant As in leaves	Essential oils/ liniment Decoction Intact
*Sonchus oleraceus* L. Asteraceae, MAHAS 6	Juwaidihia, juadheedh	Aerial parts	Bronchitis, pertussis, ophthalmia	Infusion/oral, liniment
*Tecoma capensis* Thunb. Lindl. Bignoniaceae, MAHAS 7	Sareemat aljeddy	Leaves Flowers	Pneumonia, enteritis, diarrhea Fragrance, tonic	Infusion/oral External use
*Jasminum sambac* Ait. Oleaceae, MAHAS 8	Yasmeen	Flowers Leaves	Ulceration, dermatosis, fever Eyewash	Infusion Infusion

Our study is not an exception in the quest for new antimicrobial and antipathogenic materials. Al-Fatimi, *et al*. [16] studied ninety crude dichloromethane, methanol and aqueous extracts from 30 medicinal plants used in Yemeni ethnomedicine. *In vitro* antimicrobial activity results indicated that 67 of these extracts (74%) showed good activity against *Staphylococcus aureus*, *Bacillus subtilis*, *Escherichia coli*, *Pseudomonas aeruginosa*, and *Micrococcus flavus*. Preliminary screening of medicinal plants in Colombia, showed *in vitro* antimicrobial properties of crude extracts of *Bidens pilosa*, *Jacaranda mimosifolia*, *Bixa orellana*, *Justicia secunda*, and *Piper pulchrum* [17]. Adonizio *et al*. [6], studying 50 South Florida medicinal plants extracts for antiquorum sensing activity using *Chromobacterium violaceum* 12427, reported excellent antiquorum sensing antipathogentic activities of six plant extracts of *Conocarpus erectus*, *Chamaecyce hypericifolia*, *Callistemon viminalis*, *Bucida burceras*, *Tetrazygia bicolor* and *Quercus virginiana*.

In this study, ethanolic crude extracts of 14 different parts of the eight plant species studied exhibited varying degrees of antimicrobial activity against four Gram-positive bacteria (*Staphylococcus aureus*, methicillin resistant *Staphylococcus aureus* (MRSA), *Bacillus subtilis* and *Bacillus cereus*), five Gram-negative bacteria (*Escherichia coli*, *Klebsiella pneumoniae*, *Salmonella typhimurium*, *Pseudomonas aeruginosa* and *Chromobacterium violaceum*) and four fungal species including *Aspergillus niger*, *Aspergillus fumigatus*, *Candida albicans* and *Candida glabrata* (Table 2).

At the same time the antipathogenic antiquorum sensing activities were observed (Table 3) with extracts of *Laurus nobilis* leaves, flowers, fruits and bark (17, 24, 15, 19 mm, respectively). *S*. *oleraceus* exhibited also prominent antiquorum sensing activity (18 mm), almost similar to *L*. *nobilis* extracts. *Rosmarinus officinalis* leaves (13 mm) and *Tecoma capensis* leaves (13 mm) had moderate antiquorum sensing activity. Weak antiquorum sensing activities were observed with extracts of *Jasminum sambac* (flowers and leaves), *Rosmarinus officinalis* (leaves), *Populus alba* (leaves) and *Populus nigra* (leaves) which all exhibited antiquorum sensing activities ranging from 8-10.5 mm.

Vattem *et al*. [1] found that the aqueous extract of *R*. *officinalis* leaves decreased violacein production by 40%, indicating its antiquorum sensing activity. Our results are in agreement with this result, where different plant extracts exhibited varying degrees of antiquorum sensing activities with the highest activities being seen for *S*. *oleraceus* and *L*. *nobilis* extracts. *T*. *capensis*, *P*. *alba* and *P*. *nigra* ethanol extracts exhibited rather weak antiquorum sensing activity, however, their butanol extract (unpublished data) recorded very strong activity which definitely validates their use in folk medicine. Gao *et al*. [18] reported that the interruption of bacterial quorum sensing by plant extracts, though poorly researched, is just an example of a potential way of controlling microbial pathogenesis. The results of our screening assays for antibacterial and antifungal activity of ethanolic extracts justify the use of such plants. Findings in this study confirm for the first time the antimicrobial activities of *P*. *alba*, *P*. *nigra*, *T*. *capensis*, and *S*. *oleraceus* (Table 2 and Table 3). However, *L*. *nobilis*, *L*. *angustifolia*, *R*. *officinalis* and *J*. *sambac* have been partially investigated for some biological activities such as antibacterial [19] antifungal [20] but not for antiquorum sensing.

Furthermore, in this study, the 14 ethanolic extracts of different plants showed varying degrees of antibacterial as well as antifungal activities against an array of Gram-positive and Gram-negative bacteria and fungi (Table 2 and Table 4). The magnitude of activity varied in terms of the type and number of bacteria and fungi tested and the part of the plant extracted. In addition it is well established that the type of the extractant has role in the bioactive fraction showing the activity. This has to do with the polarity of these extractants where in our case ethanol is highly polar which probably means getting different profile in the activity if other extractants of different polarity were used.

**Table 2:** Antimicrobial activity (mm inhibition zones diameter) of the ethanol plants extracts at 3 mg/disc.

Name	Part used	S.a	MRSA	B.s	B.c	K.p	S.t	E.c	P.a	C.v	C.a	C.g	A.n	A.f
T.c	Leaves	10 ±1.4	8.0±1.4	19±0.7	9.0±1.4	10±1.4	9.0±1.4	9.5±0.7	7.5±0.7	8.5±0.7	ND	ND	14±0.7	15±0.7
	Flowers	8.0±1.4	12.5±0.7	10±1.4	7.5±0.7	8.0±1.4	10±1.4	8.0±1.4	6.5±0.7	ND	15±0.7	14±0.7	13±0.7	15±0.7
La	Flowers	9.5±0.7	15±0.7	12.5±0.7	10±1.4	10.5±0.7	7.5±0.7	10±1.4	ND	7.0±0.7	11±0.0	9.0±1.4	15±0.7	15±0.7
R	Leaves	10±1.4	12±2.1	12±2.1	13±0.7	ND	13±0.7	10±1.4	ND	7.5±0.7	16.5±0.7	12±2.1	16±0.7	17.5±0.7
	Flowers	14.5±0.7	13±0.7	14±0.7	14±0.7	10±1.4	11±0.0	10±1.4	9.0±1.4	8.0±1.4	11±0.0	12±2.1	12±2.1	10±1.4
J	Leaves	13±0.7	13±0.7	10±1.4	11±0.0	13±0.7	15±0.7	11±0.0	7.5±0.7	8.0±1.4	11±0.0	10±1.4	9.0±1.4	9.5±0.7
	Flowers	10±1.4	16±0.7	11±0.0	11.5±0.7	13±0.7	9.0±1.4	11.5±0.7	ND	7.5±0.7	13±0.7	12±2.1	11±0.0	11±0.0
P.a	Leaves	9.0±1.4	8.0±1.4	7.5±0.7	10.5±0.7	10±1.4	ND	7.5±0.7	ND	8.0±1.4	9.0±1.4	11±0.0	11±0.0	10±1.4
P.n	Leaves	8.0±1.4	10±1.4	8.0±1.4	10.5±0.7	7.5±0.7	ND	10±1.4	ND	6.5±0.7	10±1.4	10.5±0.7	13±0.7	11±0.0
S	Aerial	11±0.0	10±1.4	15±0.7	ND	12±2.1	6.5±0.7	9.0±1.4	10±1.4	10±1.4	9.5±0.7	8.0±1.4	10.5±0.7	11±0.0
L.n	Fruits	9.5±0.7	12±2.1	9.5±0.7	14.5±0.7	10±1.4	9.0±1.4	9.5±0.7	8.0±1.4	9.0±1.4	9.0±1.4	9.5±0.7	13±0.7	11±0.0
	Leaves	15±0.7	16±0.7	22±0.0	14±0.7	18±0.7	10±1.4	14.5±0.7	17±0.7	14±0.7	12.5±0.7	11±0.0	25±0.7	21±0.7
	Flowers	13±0.7	11±0.0	11±0.0	11±0.0	11±0.0	10±1.4	8.0±1.4	11±0.0	15±0.7	11.5±0.7	12.5±0.7	17±0.0	19±0.7
	Bark	16±0.7	18±0.7	13.5±0.7	18±0.7	7.0±0.7	9.0±1.4	6.5±0.7	6.5±0.7	12±2.1	11±0.0	11±0.0	17±0.0	18±0.7
P	-	39±1.4	33±1.4	26±1.4	30±2.1	-	-	-	-	-	-	-	-	-
T	-	-	-	-	-	28±1.4	26±1.4	22±0.0	15±0.7	40±2.8	-	-	-	-
N	-	-	-	-	-	-	-	-	-	-	26±1.4	27±1.4	22±0.0	23±0.7

^a ^Expressed as the x ± S.D. mean diameter (mm) of growth inhibition zone and S.D. plant species: *Tecoma capensis* (T.c); *Lavandula angustifolia* (La); *Rosmarinus officinalis* (R); *Jasminum sambac* (J); *Populus alba* (Pa); *populus nigra* (Pn ); *Sonchus oleraceus* (S); *Laurus nobilis* (Ln). Microbial species: *Staphylococcus aureus* (S.a); methicillin resistant *S. aureus* (MRSA); *Bacillus subtilis* (B.s); *Bacillus cereus* (B.c); *Escherichia coli* (E.c); *Klebsiella pneumoniae* (K.p); *Salmonella typhimurium* (S.t); *Pseudomonas aeruginosa* (P.a); *Chromobacterium violaceum* (C.v); *Candida albicans* (C.a); *Candida glabrata* (C.g); *Aspergillus niger* (A.n); *Aspergillus fumigatus* (A.f). Standard antibiotic: penicillin G [P]; tetracycline [T]; nystatin [N]. ND: no detected activity at this concentration. 30% DMSO as a negative control did not show any inhibitory activity.

**Table 3:** Diameter (mm) of antiquorum sensing activity of different ethanolic plants extracts at the concentration of 3 mg per disc. Tetracycline at 30 µg/disc was used as a positive control of total microbial growth inhibition.

Plant name	Part used	Zone of QS Inhibition (mm)^a^
*Tecoma capensis*	Flowers Leaves	11±1.0 13±0.5
*Lavandula angustifolia*	Flowers	9.5±0.5
*Rosmarinus officinalis*	Flowers Leaves	9.0±0.5 13±0.5
*Jasminum sambac*	Leaves Flowers	10.5±0.9 9.0±0.5
*Populus nigra*	Leaves	8.5±0.5
*Populus alba*	Leaves	10±1.0
*Sonchus oleraceus*	Aerial	18±0.5
*Laurus nobilis*	Leaves Flowers Fruits Bark	17.5±0.5 24±0.9 15±0.9 19±0.5

^a ^Expressed as the x±S.D. mean diameter (mm) of quorum sensing inhibition zone and S.D.

**Table 4:** Minimum inhibitory concentration (MIC) in mg/disc for different ethanol plants extracts.

Plants	MIC (mg/disc)												
	S.a	MRS.a.	B.s	B.c	E.c	K.p	S.t	P.a	C.v	C.a	C.g	A.n	A.f
*Laurus nobilis*													
Fruit	0.75	0.5	0.75	0.5	1.5	1.5	1.5	2.0	1.0	0.75	1.0	0.5	0.5
Leaves	0.5	0.5	0.5	0.5	0.5	0.5	1.0	0.5	0.5	1.2	0.75	0.5	0.5
Bark	0.5	0.5	0.5	0.5	3.0	2.4	1.0	3.0	0.5	0.5	0.5	0.5	0.5
Flower	0.5	1.0	1.0	0.5	2.0	1.0	1.2	1.0	0.5	0.5	1.0	0.5	0.75
*Jasminum sambac*													
Flower	0.5	0.5	0.5	0.5	0.5	0.5	1.0	ND	2.4	0.5	0.5	0.5	0.5
Leaves	0.5	0.5	0.5	0.5	0.5	0.5	0.5	2.0	1.5	0.75	0.5	1.2	2.0
*Rosmarinus officinalis*										
Flower	1.2	1.2	0.75	0.5	1.5	ND	1.0	ND	3.0	0.75	0.5	0.5	1.2
Leaves	0.5	0.5	0.5	0.5	1.0	1.0	1.0	2.0	2.4	0.5	1.0	1.0	0.75
*Tecoma capensis*													
Flower	2.4	0.5	0.5	2.0	2.0	1.5	1.2	3.0	3.0	0.5	0.5	0.5	0.5
Leaves	1.0	1.5	0.5	1.0	1.0	1.0	2.0	2.4	2.0	ND	3.0	0.75	0.5
*Sonchus oleraceus*													
Aerial	1.2	**0.75**	0.5	ND	1.2	1.2	3.0	1.5	2.0	2.0	1.5	2.0	2.0
*Lavandula angustifolia*													
Flower	1.2	0.5	0.5	0.5	0.5	0.5	2.4	ND	3.0	1.2	0.5	0.5	0.75
*Populus nigra*													
Leaves	1.5	0.5	1.5	0.5	0.5	2.0	ND	ND	3.0	0.5	0.5	0.5	0.75
*Populus alba*													
Leaves	1.0	1.5	2.4	0.5	2.0	0.5	ND	ND	2.0	0.5	1.0	0.5	0.5
Penicillin	1.3^-4^	**6.0^-5^**	3.0^-4^	**9.0^-4^**	NT	NT	NT	NT	NT	NT	NT	NT	NT
Tetracycline	NT	NT	NT	NT	**6.0^-4^**	**6.0^-4^**	**6.0^-4^**	**9.0^-4^**	**6.0^-4^**	NT	NT	NT	NT

Microbial species: *Staphylococcus aureus* (S.a); methicillin resistant *S. aureus* (MRSA); *Bacillus subtilis* (B.s); *Bacillus cereus* (B.c); *Escherichia coli* (E.c); *Klebsiella pneumonia* (K.p); *Salmonella typhimurium* (S.t); *Pseudomonas aeruginosa* (P.a); *Chromobacterium violaceum* (C.v); *Candida albicans* (C.a); *Candida glabrata* (C.g); *Aspergillus niger* (A.n); *Aspergillus fumigatus* (A.f). 30% DMSO as a negative control did not show any inhibitory activity. ND: no detected activity at this concentration. NT: not tested. Mystatin MIC was not determined.

Erturk [21] reported that the ethanolic extracts of *L*. *nobilis* leaves showed higher inhibitory activity against *A*. *niger* and *Candida albicans* than the standard antifungal nystatin. Our results show a similar trend, whereby ethanolic extracts of *L*. *nobilis* leaves, for example, showed a broad spectrum antibacterial activity at 3 mg per disc against Gram-negative *P*. *aeruginosa* (17 mm inhibition zone) compared with tetracycline (15 mm), and exhibited a superior antifungal activity against *A*. *niger* (25 mm) and *A*. *fumigatus* (21 mm) compared to nystatin (22, 23 mm, respectively) (Table 2).

Joy and Raja [19] reported that ethanolic extracts of the callus of *J*. *sambac* exhibited antibacterial activity against both Gram-positive *S*. *aureus* and Gram-negative *S*. *typhi* and *P*. *mirabilis*. In this study *J*. *sambac* (flowers and leaves) extracts were very active (>15 mm inhibition zone) against Gram-positive methicillin resistant *S*. *aureus*, *B*. *subtilis*, as well as against Gram-negative *E*. *coli*, *S*. *typhimurium* and *K*. *pneumoniae* and fungi, including the filamentous *A*. *niger*, *A*. *fumigates*, and the yeasts *Candida albicans* and *Candida glabrata* (Table 2 and Table 4). The antimicrobial activity profile of all plant extracts tested (Table 2 and Table 4) indicates for the first time the antimicrobial activity of *P*. *alba*, *P*. *nigra* and *T*. *capensis*. This profile revealed that these plant extracts have a strong antifungal activity (11-15 mm) relative to nystatin (22 mm) and a moderate to good activity against both Gram-positive and negative bacteria when tested at 3 mg per disc. The lowest minimum inhibitory concentration recorded in this study was 0.5 mg per disc and it was for several bacteria and fungi among these tested and few microorganisms exhibited higher MIC's (2-3 mg per disc; Table 4). These MIC values for the different bacteria and fungi tested, though relatively high (0.5-3 mg per disc), are definitely demonstrative of the potential clinical use [13,22], knowing that these are just crude extracts of uncertain composition with components that can have synergistic or antagonistic effects [5,6,20] which require further investigation. Finally, these results clearly indicate that the different plant parts screened possess substantial antiquorum, antibacterial and antifungal activity. This agrees with and validates the use of these plants in the traditional pharmacopia of Jordan.

## Conclusions

In the quest to control bacterial and fungal infections ethanolic extracts of different parts of *T*. *capensis*, *S*. *oleraceus*, *P*. *alba*, *P*. *nigra*, *J*. *sambac*, *R*. *officinalis*, *L*. *angustifolia* and *L*. *nobilis* show great potential as a source of microbial growth and quorum sensing inhibitors. Further studies are needed to determine and characterize the active ingredients, especially of those plants whose activity is reported herein for the first time.

## Experimental

### Plant material

Leaves, flowers, fruits and stem bark of the plants (Table 1) were collected whenever available during March-November 2007, from Jubaiha, 10 Km North of Amman, Jordan. The plants were authenticated by Prof. A. El-Oqlah, a plant taxonomist at Yarmouk University, Irbid, Jordan, and Prof.D.El-esawi of the University of Jordan, Amman, Jordan.Voucher specimens (Table 1) were deposited in the Department of Biological Sciences, University of Jordan, Amman. The collected plant material were air-dried under shade at room temperature, milled into a fine powder using an electric mill (Breams, UK) and were stored in an airtight plastic sampling bags for later analysis.

### Plant extraction

The air-dried plant materials were separately extracted twice at room temperature with ethanol 95% (500 mL/100 g of plant material each run). The final ethanol extract of each plant part was filtered using filter paper (Whatman) and evaporated under vacuum at 40 °C using a rotary vacuum evaporator (Buchi R-215, Switzerland). The resultant residues from the different plants parts were further fractionated according to Mahasneh [12] and were stored at -20 °C for further analysis.

### Antimicrobial assays

*Microbial cultures*: Microorganisms used for the determination of antimicrobial activities of the different extracts included Gram-positive bacteria: *Staphylococcus aureus*, methicillin resistant *Staphylococcus aureus* (MRSA clinical isolate), *Bacillus cereus* (Toxigenic strain) and *Bacillus subtilis*. Gram-negative bacteria: *Salmonella typhimurium* (ATCC 14028), *Klebsiella pneumonia* (ATCC 10031), *Escherichia coli*, *Pseudomonas aeruginosa*, *Chromobacterium violaceum*, and both filamentous fungi; *Aspergillus fumigatus* (Clinical isolate), *Aspergillus niger* (ATCC 16404) and yeasts: *Candida glabrata* (Clinical isolate), and *Candida albicans* (ATCC 10231). All microbial strains were obtained from our stock cultures in the Department of Biological Sciences, University of Jordan, Amman.

The different bacterial strains were maintained onto nutrient agar slants at 4 °C. For antibacterial activity testing, bacterial cultures were prepared into a tube containing nutrient broth (Idg, England, 5 mL) and incubated at 37 °C for an overnight. The optical densities of the cultures were adjusted to match 0.5 McFarland standard *i.e. ,* 1 × 10^8 ^colony forming units per mL. Cultures of filamentous fungi and yeasts were grown on malt extract agar (Merck, Germany) at 28 °C and maintained at 4 °C onto malt extract agar slants.

*Antimicrobial activity testing*: Different extracts of the perspective plants parts were dissolved in DMSO, membrane filter (pore size 0.45 µm) sterilized and tested for antimicrobial activity using the agar diffusion method. Sterile 6 mm diameter filter paper discs were impregnated with 3 mg of the sterile appropriate extract and were placed in duplicates onto Muller-Hinton agar (Oxoid, England) plates for bacteria and malt extract agar for yeast and filamentous fungi. These plates were earlier surface inoculated separately with 100 µL of either freshly prepared bacteria, fungal spores of yeast cells suspension (Ca. 10^8 ^CFU/mL). The plates were kept for 2 h at 4 °C to facilitate diffusion of the extracts into the agar and were then incubated for 24 h at 37 °C (for bacteria) or for 48-72 h at 28 °C (for fungi). Inhibition zone diameters around each of the discs were measured and recorded at the end of the incubation time. Reported inhibition zones are the average calculated from at least two replicates. Separate negative control discs contained either sterile DMSO or ethanol. For comparative purposes, standard antibacterials penicillin G (10 U/disc), tetracycline (30 µg/disc) and antifungal nystatin (100 µg/disc) (Oxoid, Basingstoke, UK) were included in the assay. Minimum inhibitory concentrations (MIC's) for the tested samples were determined by the agar diffusion assay [23] using media and incubation temperature as recommended for both bacteria and fungi. Negative controls of DMSO alone were included as well as positive controls of the standard antibiotics penicillin and tetracycline. MIC was defined as the lowest concentration of the extract that totally inhibited the growth of the tested microorganisms.

*Antiquorum sensing assays*: The *Chromobacterium violaceum* quorum sensing system was used for this assay. Quorum sensing (QS) in this wild type strain of bacteria is known [24] to control production of violacein (a purple pigment) due to the production and in response to autoinducer molecules such as C_6_-acyl homoserine lactones and C_4_-acyl homoserine lactones. *C*. *violaceum* strain ATCC 12427 was kindly provided by Professor Robert McClean of the Department of Biology, Texas State University-San Marcos, TX, USA. It was routinely cultured aerobically in Luria Bertani agar (Merck, Germany) at 30 °C prior to testing. Cultures were maintained at room temperature for daily use. Stock cultures were kept at -20 °C in Luria Bertani broth (4 mL) supplemented with 25% (v/v) sterile glycerol as a cryoprotectant.

The disc diffusion method was employed to detect the anti-QS activity of the different plant extracts. In this test, bacterial growth inhibition would result in a clear halo around the disc, while a positive result of quorum sensing inhibition is exhibited by a turbid halo harboring pigmentless bacterial cells of the *Chromobacterium violaceum* 12427 monitor strain. Cultures of *Chromobacterium violaceum* 12427 were prepared by growing bacteria in Luria Bertani broth (Merck, Germany) and incubated for 16-18 h in an orbital incubator (Labtech, Korea) running at 30 °C and 150 rpm. Cultures were then adjusted to 0.5 McFarland standard (Ca. 10^8 ^CFU/mL). Discs used (6 mm diameter) were made of sterile filter paper (Whatman, UK) and were impregnated with the appropriate concentration of the extract. Ethanol extracts of different plants were dissolved in sterile DMSO. These discs were dried and were then transferred in triplicates per concentration onto *Chromobacterium violaceum* inoculated (0.1 mL per plates) Luria Bertani plates which were then incubated at 30 °C for 24 h after which results were recorded. To ensure the sterility of the sample and to minimize any introduction of exogenous anti QS compounds, extracts were membrane (0.45 µm) sterilized and were tested for microbial contamination before the anti QS assay by streaking onto Luria Bertani agar (LB) plates and incubation at 37 °C for overnight. These were positively controlled by observing bacterial growth inhibition zones by standard tetracycline discs (30 µg per disc). 

### Statistical analysis

Antimicrobial activity values are expressed as mean ± S.D. The software Graph Pad Prism was employed for calculations.
